# Severe hypoglycemia in propranolol treatment for infantile hemangiomas

**DOI:** 10.1111/ped.15278

**Published:** 2022-08-16

**Authors:** Akira Morimoto, Michio Ozeki, Satoru Sasaki, Naoko Baba, Yoshihiro Kuwano, Tsuyoshi Kaneko

**Affiliations:** ^1^ Department of Pediatrics Jichi Medical University School of Medicine Shimotsuke Japan; ^2^ Division of Pediatrics Showa Inan General Hospital Komagane Japan; ^3^ Department of Pediatrics Gifu University Graduate School of Medicine Gifu Japan; ^4^ Center for Vascular Anomalies KKR Sapporo Medical Center Tonan Hospital Sapporo Japan; ^5^ Department of Dermatology Kanagawa Children's Medical Center Yokohama Japan; ^6^ Department of Dermatology Mizonokuchi Hospital, Teikyo University School of Medicine Kawasaki Japan; ^7^ Department of Plastic and Reconstructive Surgery National Center for Child Health and Development Tokyo Japan

**Keywords:** β‐adrenergic receptor blocker, hypoglycemia convulsion, infantile hemangioma, propranolol

## Abstract

**Background:**

Infantile hemangioma (IH), formerly termed strawberry hemangioma, is a benign vascular tumor caused by capillary endothelial cell proliferation. The tumor regresses after 1 year of age, but sequelae occur in approximately half of the patients without systemic treatment. Propranolol (PPL) is currently the first‐line therapeutic agent in Japan as well as in Western countries. It is not commonly known that PPL may induce severe hypoglycemia, in addition to cardiovascular and respiratory side effects.

**Methods:**

We retrospectively analyzed patients with severe PPL‐induced hypoglycemia in the 3 years since the launch of Hemangiol®, a PPL preparation specific for IH, in Japan in 2016.

**Results:**

The incidence of severe hypoglycemia and of hypoglycemic convulsions following PPL treatment was estimated to be 0.54% and 0.35%, respectively. The incidence of hypoglycemic convulsions appeared to be higher in Japan than in Western countries. Severe hypoglycemia was common in infants aged >1 year, when PPL was used for ≥6 months. Severe hypoglycemia often develops from 05:00 a.m. to 09:00 a.m. and is frequently associated with prolonged periods of fasting, poor feeding, or poor physical conditions.

**Conclusion:**

To avoid the risk of hypoglycemia, the treatment should be initiated by 6 months of age during the proliferative phase at the latest, and should not be extended indiscriminately beyond 1 year of age. Guardians should be advised not to administer PPL on an empty stomach, in the presence of poor feeding, or who are in poor physical condition, not to prolong fasting after PPL administration, and to monitor the child's condition immediately after he or she wakes up.

Infantile hemangioma (IH), traditionally referred to as strawberry hemangioma, is now classified as a benign vascular tumor in the International Society for the Study of Vascular Anomalies (ISSVA) classification.^1^ The condition exhibits a characteristic clinical course. It may be unnoticed at birth. The lesion generally grows rapidly 2–3 months after birth, stabilizes from 6 months to 12 months of age, and regresses after 1 year of age.^2^ Without systemic treatment, approximately half of the patients with IH suffer from sequelae such as telangiectasia, fibrolipoma, and skin atrophy.^3^ Patients with IH have traditionally been treated with lasers or corticosteroids, but these treatment options have not been sufficiently effective.

In 2008, the high efficacy of propranolol (PPL), a non‐selective β‐adrenergic receptor blocker, for IH was reported.^4^ In 2014, PPL oral solution for infants was developed by Pierre Fabre Dermatologie (PFD) in France.^5^ Subsequently, in 2016, Maruho Co., Ltd. in Japan obtained approval for the manufacture and sale of an IH‐specific PPL preparation – Hemangiol® syrup 0.375%(Hemangiol) – following a phase III clinical trial.^6^ As PPL is highly effective for IH in the proliferative phase, it is recommended as the first‐line treatment for IH with a risk of organ dysfunction and cosmetic problems both in Western countries and Japan.^7^, ^8^

It has been known for a long time that PPL can cause adverse events affecting the metabolic system (hypoglycemia), in addition to the cardiovascular system (bradycardia, atrioventricular block) and respiratory system (bronchospasm).^9^ Cardiovascular adverse events usually appear at the initiation of treatment^10^ and can be managed by starting oral administration under medical supervision. Respiratory adverse events can also be prevented by not prescribing PPL to patients with a history of asthma or by discontinuing oral administration of the drug during respiratory infections. In contrast, hypoglycemia appears unexpectedly, even after long‐term oral administration.^10^ Severe hypoglycemia sometimes causes serious neurological sequelae such as developmental retardation and epilepsy. Propranolol has been used for many years for heart disease but hypoglycemia has been reported only in a few cases associated with fasting at surgery.^11^ On the other hand, PPL‐induced hypoglycemia has become a more frequent and serious problem among patients with IH treated with PPL.^12^ The reasons may be because the prevalence of IH is relatively high in infants, up to 4–5%,^2^ and PPL is often prescribed to patients with IH by physicians who are less familiar with the use of PPL. We now know that PPL‐induced hypoglycemia in patients with IH develops in both newborns and infants, is not dose‐dependent (develops even at relatively low doses of 1.25 to 2.0 mg/kg/day), and develops at unexpected times, but more commonly in the presence of poor feeding or infections.^12^, ^13^

In Japan, information on adverse events associated with Hemangiol is provided by the manufacturer, Maruho (Osaka, Japan), through the product information document and explanatory materials for guardians. However, hypoglycemia associated with the oral administration of Hemangiol is not fully understood, and cases of severe hypoglycemia continue to be reported in Japan.

We analyzed the frequency and factors associated with Hemangiol‐induced hypoglycemia in patients with IH in Japan, based on the adverse event reports.

## Methods

We analyzed the adverse event reports of severe hypoglycemia associated with the treatment of Hemangiol for IH that developed 3 years after the launch of Hemangiol in Japan (September 16, 2016, to September 15, 2019). Hypoglycemia events corresponding to (i‐iV) defined by the Pharmaceuticals and Medical Devices Agency (PMDA) was determined by the physician to be severe hypoglycemia: (i) death; (ii) disability; (iii) events that may lead to death or disability; (iv) events requiring hospitalization or extension of hospital stay for treatment; (v) events corresponding to (i—iv); or (vi) inducing congenital illness or abnormality in later generations. The deidentified adverse event reports collected by Maruho from physicians for reporting to the PMDA were provided by Maruho for this study. The cumulative number and age ratio of patients taking Hemangiol were estimated by Maruho from the prescription data. The times of onset of hypoglycemia were divided into the following seven time zones: A 05:00–07:00 a.m., B, 07:00–09:00 a.m.; C, 09:00–11:00 a.m.; D, 11:00 a.m.–13:00 p.m.; E, 13:00–15:00 p.m.; F, 15:00–17:00 p.m.; G, 17:00–19:00 p.m. Prolongation of fasting was defined as 10 h or more. This study was approved by the review board of Showa Inan General Hospital (No.2021‐07).

## Results

We identified 28 patients with severe hypoglycemia. The characteristics of these patients are shown in Table 1. The median minimal blood glucose level of these patients (except for four patients with missing data) was 20 mg/dL (range, 10–68 mg/dL). Hypoglycemic convulsions were observed in 18 of 28 patients (64%). Since the cumulative number of patients prescribed Hemangiol during the 3 years was estimated to be 5,164 patients, the incidence of severe hypoglycemia and hypoglycemic convulsions was estimated to be 0.54% and 0.35%, respectively.

**Table 1:** Characteristics of patients with severe hypoglycemia during treatment with Hemangiol

UPN	Sex	Age at developing hypoglycemia (months)	Age at the initiation of taking Hemangiol (months)	Duration taking Hemangiol (months)	Body weight (kg)	Dose of Hemangiol		Time zone *	Minimum blood glucose levels (mg/dL)	Convulsions	Risk factors for hypoglycemia				Neurological sequelae
						(mg/day)	(mg/kg/day)				Prolongation of fasting ** (hours)	Poor feeding	Poor physical condition	Others	
1	F	2	2	0.1	2.3	2.3	1.0	ND	35	−	ND	−		LBW	−
2	M	3	3	0.1	6.9	6.8	1.0	B	39	−	−	−	−		−
3	F	7	7	0.1	ND	ND	1.0	B	43	−	+ (14.5)	−	−		−
4	M	9	4	5	8.4	25.5	3.0	A	<20	+	+ (11.0)	+	−		−
5	F	10	3	7	7.6	22.5	3.0	B	20	+	ND	+	URI		−
6	F	11	4	7	ND	ND	ND	B	13	+	+ (16.5)	+	−		−
7	F	11	8	3	9.9	28.5	2.9	B	20	+	ND	+	URI		−
8	F	13	2	11	ND	ND	2.0	B.	17	+	+ (13.0)	−	−		−
9	M	13	1	12	10.0	30.0	3.0	G	12	+	ND	+	Diarrhea		−
10	F	13	1	12	ND	ND	3.0	A	40	+	ND	−	URI		−
11	F	14	4	10	9.6	28.5	3.0	D	ND	−	ND	−	−		−
12	M	14	9	5	9.0	27.0	3.0	C	16	+	+ (16.5)	−	−		−
13	F	14	3	11	7.5	15.0	2.0	E	45	−	ND	−	Diarrhea		−
14	M	15	1	14	10.3	30.8	3.0	B	29	+	ND	+	−		−
15	M	15	ND	ND	ND	ND	3.0	ND	ND	−	ND	+	−		−
16	F	16	4	12	9.4	28.5	3.0	B	14	+	+ (12.0)	+	−		−
17	F	16	8	8	9.8	12.8	1.3	B	11	+	+ (12.0)	−	Diarrhea		−
18	M	18	2	16	9.0	27.0	3.0	B	19	+	+ (12.0)	−	−		−
19	M	19	10	9	ND	ND	ND	ND	36	−	ND	+	−		−
20	F	20	5	15	12.0	24.0	2.0	A	24	+	+ (10.0)	+	Viral infection		−
21	F	21	6	15	10.0	30.0	3.0	B	15	+	ND	+	Fever		−
22	F	22	17	5	ND	ND	3.0	A	ND	−	ND	+	Fever		−
23	F	23	22	1	ND	ND	3.0	ND	ND	+	ND	+	−		−
24	F	24	11	13	ND	ND	2.0	B	16	+	+ (13.5)	+	−		Epilepsy
25	M	13	6	7	10.0	30.0	3.0	B	20	+	+ (13.0)	−	−		−
26	F	14	3	11	10.7	20.3	1.9	B	16	+	+ (12.0)	−	−		−
27	F	17	5	12	12.0	36.0	3.0	ND	68	−	ND	+	URI		−
28	M	43	ND	ND	ND	ND	3.0	ND	10	−	ND	+	ND		−

* : A 05:00–07:00 a.m., B, 07:00–09:00 a.m.; C, 09:00–11:00 a.m.; D, 11:00 a.m.–13:00 p.m.; E, 13:00–15:00 p.m.; F, 15:00–17:00 p.m.; G, 17:00–19:00 p.m.

** Defined as 10 h or more.

Hemangiol, Hemangiol^®^ syrup 0.375%; LBW. Low birthweight; ND, no data; URI, upper respiratory infection.

**Fig. 1:**
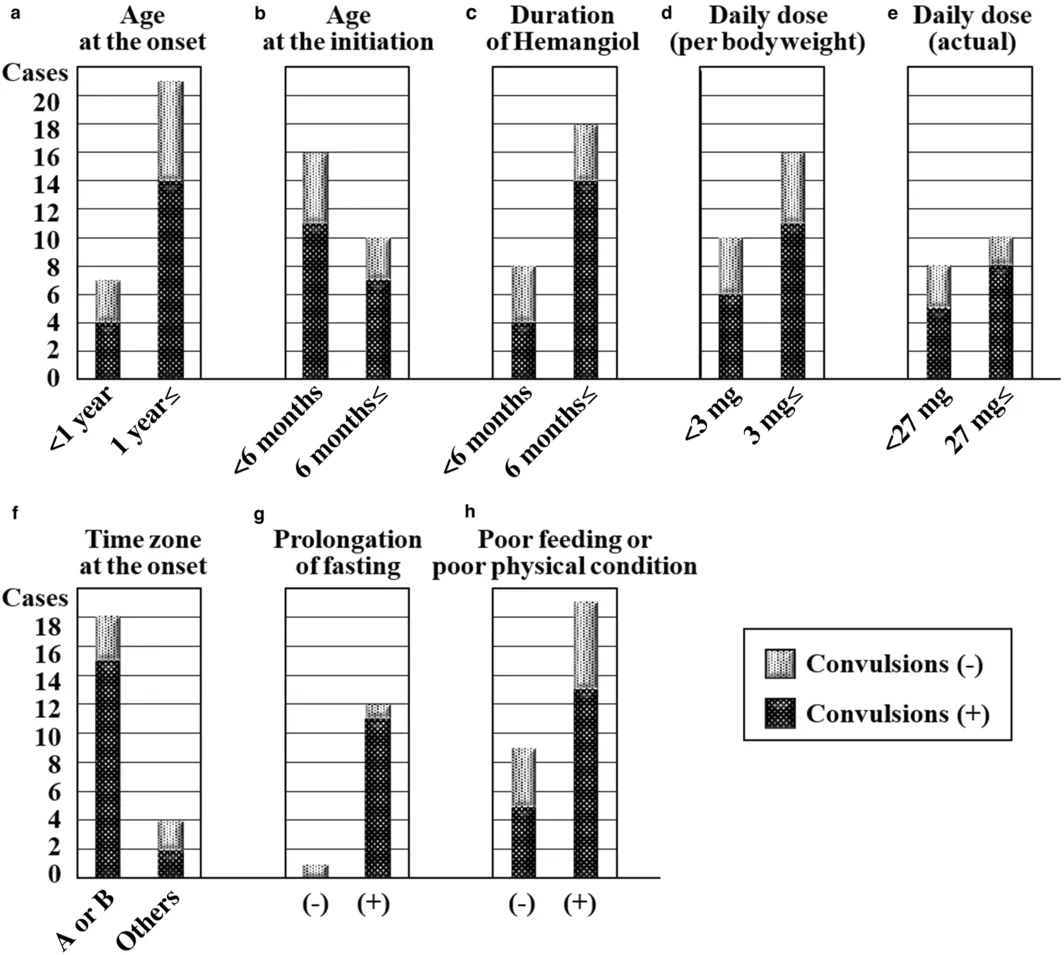
Risk factors for developing severe hypoglycemia and hypoglycemic convulsions. (a) Severe hypoglycemia was more common in patients aged ≥1 year, especially hypoglycemic convulsions. (b) Many patients with severe hypoglycemia commenced treatment with Hemangiol® syrup 0.375% (Hemangiol) after 6 months of age. (c) The duration of Hemangiol use was 6 months or longer in the majority of patients with severe hypoglycemia. (d) Severe hypoglycemia developed even at doses <3 mg/kg/day. (e) Most patients with hypoglycemic convulsions were taking Hemangiol at a daily dose of 27 mg or more. (f) Severe hypoglycemia developed most frequently in time zone A or B (from 5 am to 9 am). (g) Most patients with severe hypoglycemia reported prolonged fasting (≥10 h). (h) Most patients with severe hypoglycemia reported poor feeding or poor physical conditions.

The median age of onset for hypoglycemia was 14 months (range, 2–43 months), and patients aged >1 year accounted for 21 of the 28 patients (75%) (Fig. 1a). Fourteen of the 18 patients with hypoglycemic convulsions (78%) were aged >1 year. Of the patients who were taking Hemangiol, the ratio of those aged >1 year was estimated to be <40% in Japan. These findings suggest that patients aged >1 year may be more likely to develop severe hypoglycemia and hypoglycemic convulsions.

The median age at which Hemangiol was commenced was 4 months (range, 1–22 months; 2 patients with missing data). Hemangiol was initiated after 6 and 12 months of age, in 10 (38%) and 2 (8%) of the 26 patients (38%), respectively (Fig. 1b). These findings indicate that patients with severe hypoglycemia started on Hemangiol treatment at an older age, with a considerable number of patients with severe hypoglycemia commencing treatment after the proliferation phase, either during the stable or regression phase.

The median treatment duration with Hemangiol when severe hypoglycemia occurred was 9.5 months (range, 0.1–16 months, data were missing from 2 patients). Only 3 of the 26 patients (12%) developed severe hypoglycemia at the initiation of treatment. The treatment duration was ≥6 months in 18 of the 26 patients (69%; Fig. 1c), and ≥1 year in 9 of the 26 patients (35%). Moreover, 14 of the 18 patients (78%) with hypoglycemic convulsions had been on Hemangiol treatment for 6 months or longer. These findings suggest that most patients with severe hypoglycemia had been treated with Hemangiol for more than the standard treatment period of 6 months. Furthermore, these patients were at higher risk of hypoglycemic convulsions.

The median Hemangiol dose per bodyweight was 3.0 mg/kg/day (range, 1.0–3.0 mg/kg/day, data missing from 2 patients). Patients who were treated with a dose of <3.0 mg/kg/day accounted for 10 of the 26 patients (38%). Five patients were treated at a dose of <2.0 mg/kg/day, and another 5 patients at 2.0–3.0 mg/kg/day (Fig. 1d). In 6 of the 17 patients with hypoglycemic convulsions (35%), the treatment dose was <3.0 mg/kg/day (data missing from 1 patient). These results indicate that severe hypoglycemia may develop even at less than the standard dose of 3.0 mg/kg/day, and the incidence of hypoglycemic convulsions did not differ depending on the dose per bodyweight.

Considering the actual treatment dose, 10 of the 18 patients (56%, data missing from 10 patients) received ≥27 mg/day (calculated at a dose of 3 mg/kg/day for a 1‐year‐old infant with standard bodyweight). Notably, 8 of the 13 patients with hypoglycemic convulsions (67%, data missing from 5 patients) had an actual dose of ≥27 mg/day (Fig. 1e).

In 18 of the 22 patients (82%, data missing from 6 patients), severe hypoglycemia occurred from 05:00 a.m. to 09:00 a.m. (time zone A or B; Fig. 1f). Of these patients, 12 of the 18 patients (67%) were aged >1 year. Prolonged fasting (≥10 h) was observed in 12 of the 13 patients (92%, data missing from 15 patients) (Fig. 1g), and 8 of the 11 patients (73%) with hypoglycemic convulsions were aged >1 year. Poor feeding was observed in 16 of the 28 patients (57%). Eleven of the 27 patients were in poor physical condition, with fever, diarrhea, or upper respiratory tract infections (41%, data missing from 1 patient). A total of 19 patients (68%) experienced either prolonged fasting or poor physical condition (Fig. 1h). Of these 19 patients, 13 of 17 patients (76%, data missing from 2 patients) had been taking Hemangiol for >6 months. One patient was a low‐birthweight infant, weighing 2.3 kg at the onset of hypoglycemia.

One patient (UPN 24) had neurological sequelae. The guardians of that patient were unaware that their infant was having convulsions for some time, and the patient was in status epilepticus, causing intractable seizures even 2 months after the onset.

## Discussion

In patients with IH treated with Hemangiol, severe hypoglycemia and hypoglycemic convulsions developed in 0.54% and 0.35%, respectively. Patients with severe hypoglycemia were more often aged >1 year, commenced Hemangiol at an older age, were treated for >6 months, and were treated at a dose of ≥27 mg/day, while complying with the standard dose of 3 mg/kg/day as they weighted over 9 kg. In addition, severe hypoglycemia often developed early in the morning in patients aged >1 year and was associated with prolonged fasting, poor feeding, or poor physical conditions. These findings suggest that patients aged >1 year, who have been completely weaned and fasted during the night are at higher risk of Hemangiol‐induced severe hypoglycemia in the early morning, and the counsel to avoid the use of Hemangiol in the presence of poor feeding or for children in poor physical condition was ignored during the long‐term use of Hemangiol.

Evidence from Western countries showed that, of the 5,862 patients who received PPL for IH, hypoglycemia was observed in 33 patients (0.56%), and hypoglycemic convulsions in four patients (0.07%).^12^ As the two cohorts did not define hypoglycemia the same way, it is not possible simply to compare the frequency of hypoglycemia in the Japanese cohort with the Western cohort. However, the incidence of hypoglycemic convulsions appeared to be higher in Japan than in Western countries.

The growth‐inhibitory effect of PPL on IH is high when PPL is initiated during the proliferation phase within 6 months of age.^14^ It has been reported that the effect of PPL on IH can also be obtained by initiating PPL therapy in the stable or regression phases; however, the efficacy rate is lower and the treatment duration longer.^15^ As such, PPL treatment should be initiated during the proliferative phase, and by 6 months after birth at the latest, preferably around 2 months of age. Fasting hypoglycemia is the predominant type of hypoglycemia that is seen in children. Infants feeding every 2–4 h rarely develop hypoglycemia, even if they have defects in glycogen breakdown, gluconeogenesis, or ketogenesis. However, infants with these defects develop hypoglycemia after 6 months of age when the feeding interval is extended to about 6 to 8 h.^16^ By initiating PPL early, dose reduction or discontinuation of Hemangiol would be possible by the time weaning and prolonged fasting at night occur, and would thus reduce the risk of hypoglycemia.

In Europe and the USA, the initiation of Hemangiol is limited to age of ≤5 months, and the treatment duration is limited to 6 months. In Japan, however, both the age for commencing Hemangiol and the treatment duration are not strictly limited. Rather the recommendations are that “in principle, it is used during the proliferative phase”, and “around 24 weeks after the initiation of treatment, evaluate the efficacy and consider the necessity of continuing treatment.” As a result, in Japan, many patients are treated with Hemangiol during the regression phase and after 1 year of age, leading to hypoglycemia due to prolonged fasting times at night. It is not clear why the incidence of hypoglycemic convulsions is higher in Japan than in Western countries, but this factor may lead to more patients with extremely low blood glucose levels in Japan, resulting in a higher incidence of hypoglycemic convulsions in Japan. It may also be necessary to limit the duration of treatment in Japan.

The dose of Hemangiol was determined per bodyweight based on a phase III clinical trial in Japan,^6^ in which all subjects were aged <1 year because it was a 24‐week study in patients aged up to 150 days. Cytochrome P450s (CYPs), the metabolic enzymes that target this drug, are expressed shortly after birth, and their expression levels increase until 1 year of age.^17^ After 1 year of age, the expression of CYPs becomes steady, and drug clearance through the liver increases in proportion to the liver's weight. It is important that the liver's weight increases in proportion to body surface area, rather than bodyweight. In turn, body surface area per bodyweight decreases with age. Taken together, the liver weight per bodyweight – that is, drug clearance per body weight – decreases after 1 year of age.^18^ If the dose for children aged >1 year is increased in terms of body weight, overdose and a higher risk of severe hypoglycemia may therefore arise. This may be the reason for the high prevalence of severe hypoglycemia in children taking Hemangiol at a dose of ≥27 mg/day. In patients aged >1 year and in the regression phase, IH does not usually worsen, even without increasing the dose in line with increased body weight. As such, the dose should be reduced rather than increased.

Poor feeding and infection are known to be a risk of developing hypoglycemia,^12^, ^13^ and this is also true in Japan. However, guardians in Japan are not well informed about this. It is fundamentally important to avoid Hemangiol on an empty stomach or when feeding is poor. This caution is clearly stated in the product information document and strongly recommended by the treatment guideline for IH in both Western countries and Japan.^7^, ^8^ Guardians should be repeatedly advised to stop giving the drug without hesitation when patients are not feeding properly or are physically unwell. It is also important to inform the guardians that IH may worsen temporarily due to drug suspension but it will improve again when treatment resumes.

The overnight fasting time becomes longer when the baby starts solid food and overnight feeding is stopped, and weaning is completed. Fasting for ≥8 h increases the risk of hypoglycemia in infants, so for patients on Hemangiol, infants aged under 6 weeks must be fed at least every 4 h, infants aged 6 weeks to 4 months at least every 5 h, and infants aged >4 months, at least every 6–8 h.^13^ At each outpatient visit, check feeding (the time of feeding baby food and the feeding situation overnight), advise against prolonged fasting of ≥8 h, and prevent prolonged fasting by feeding before going to bed and immediately after waking up. Hypoglycemia frequently occurs in the early morning, so it is important to advise the guardians to check their infants' condition immediately after waking up. For example, they should check whether the infant is as active as usual. In our study, some guardians of patients with hypoglycemic convulsions did notice that their infants were not doing well and were unusually sleepy, the infants were left in bed. Subsequently, hypoglycemic convulsions occurred. It is therefore important to educate guardians that “not doing well” or “being sleepy” may be a sign of hypoglycemia. Moreover, guardians should be advised to feed their infants some sugar, when these signs are first observed.

Our case series included one patient with low birthweight. Preterm infants have a higher risk of hypoglycemia due to higher glucose utilization during fasting and a lower glycogen storage.^19^ Even the standard dose would be excessive for premature infants due to the immaturity of both the hepatic metabolism and renal excretion of PPL. The product information document for Hemangiol in Japan states that patients aged <5 weeks should be treated with caution. For infants younger than 5 weeks (corrected gestational age), Hemangiol should be limited to patients with life‐threatening lesions, and the initial dose must be adjusted to less than half the usual dose, i.e. ≤0.5 mg/kg/day.

### Conclusion

Although Hemangiol is highly effective for IH during the proliferative phase, it can cause adverse events, including severe hypoglycemia. The incidence of hypoglycemic convulsions appears to be higher in Japan than in Western countries. Hypoglycemic convulsions are more common in patients aged >1 year, and in the early morning, associated with prolonged overnight fasting. The risk of adverse events can be reduced by the guardians taking sufficient precautions when giving Hemangiol. The most important point for the physicians to keep in mind is that the treatment should be initiated by 6 months of age at the latest, preferably around 2 months of age, and should not be extended indiscriminately beyond 1 year of age. By increasing awareness, Hemangiol can be used more safely for patients with IH.

## Disclosure

A.M., M.O., S.S., N.B., Y.K., and T.K. received patient data and funding for the present manuscript from Maruho Co., Ltd. A.M., M.O., S.S., N.B., and Y.K. received lecture fees from Maruho Co., Ltd. M.O., S.S., N.B., Y.K., and T.K. received research funding from Maruho Co., Ltd. A.M., M.O., S.S., N.B., Y.K., and T.K. received consulting fee from Maruho Co., Ltd. A.M., M.O., S.S., N.B., Y.K., and T.K. were members of the advisory board on the proper use of Hemangiol provided by Maruho Co., Ltd.

## Author contributions

A.M., O.M., S.S., N.B., Y.K., and T.K. designed the study and analyzed the data without the intervention of Maruho Co., Ltd. A.M. wrote the manuscript. All authors read and approved the final manuscript.
